# Thyroglossal Duct Carcinoma Originating in the Hyoid Bone

**DOI:** 10.1155/2019/3067346

**Published:** 2019-07-01

**Authors:** Naoki Kunitomo, Hiroyuki Fujii, Akifumi Fujita, Yumiko Hamano, Minako Takanosawa, Hideharu Sugimoto

**Affiliations:** ^1^Department of Radiology, Jichi Medical University, School of Medicine, 3311-1 Yakushiji, Shimotsuke, Tochigi 329-0498, Japan; ^2^Department of Otolaryngology-Head and Neck Surgery, Jichi Medical University, School of Medicine, 3311-1 Yakushiji, Shimotsuke, Tochigi 329-0498, Japan

## Abstract

Thyroglossal duct (TGD) carcinoma is a rare malignant tumor arising from remnants of thyroid tissue or the wall of the duct and generally occurs along the anatomic course of the TGD. TGD carcinoma originating in the hyoid bone is extremely rare but can occur since the TGD penetrates the hyoid bone on rare occasions. This report describes the case of a 30-year-old man with TGD carcinoma originating in the hyoid bone. Computed tomography demonstrated a mass in the hyoid bone that expanded the cortical bone of the hyoid. The mass had a central solid component with calcification and a marginal cystic component. When we encounter a calcified mass in the hyoid bone, we should consider TGD carcinoma among the differential diagnoses.

## 1. Introduction

Thyroglossal duct (TGD) carcinoma is a rare malignant tumor arising from remnants of thyroid tissue or the wall of the TGD. This neoplasm may occur within a thyroglossal duct cyst on some occasions. TGD carcinoma generally occurs along the anatomic course of the TGD, which begins at the foramen cecum, loops around the hyoid bone, and descends anteriorly to the thyrohyoid membrane, uncommonly penetrating the hyoid bone [1]. TGD carcinoma originating in the hyoid bone is extremely rare, with only one case previously reported in the English-language literature [2]. Herein, we report the case of a 30-year-old man with TGD carcinoma originating in the hyoid bone.

## 2. Case Report

A 30-year-old man presented to our hospital complaining of a six-month history of progressive neck swelling and sore throat. He had no pertinent past medical history. Physical examination revealed a 4-cm, hard mass in the right anterior neck. Results of thyroid functional tests, such as serum thyroxine (T4), free triiodothyronine (T3), and thyroid stimulating hormone (TSH), were within normal limits. Other laboratory findings were also unremarkable.

**Figure 1:**
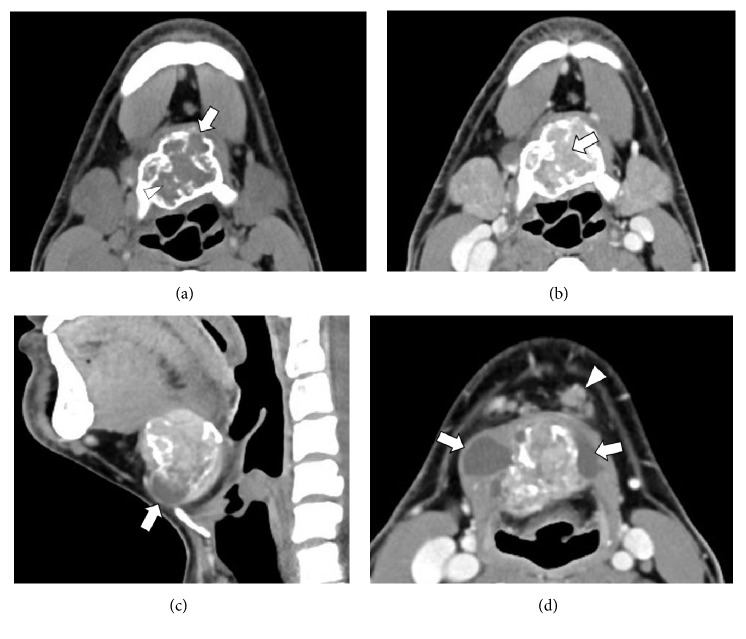
CT of the neck. (a) Axial noncontrast CT at the level of the hyoid bone shows a well-defined, low-density mass expanding the cortex of the hyoid bone with partial bone destruction (arrow). Punctate calcifications are seen within the mass (arrowhead). (b) Axial contrast-enhanced CT at the same level as (a) shows marked enhancement of the solid component (arrow). (c, d) Sagittal at the midline (c) and axial at the infrahyoid level (d) images of contrast-enhanced CT show the mass consists of a solid component and a marginal cystic component (arrows). Left submental lymphadenopathy is also seen (arrowhead).

**Figure 2:**
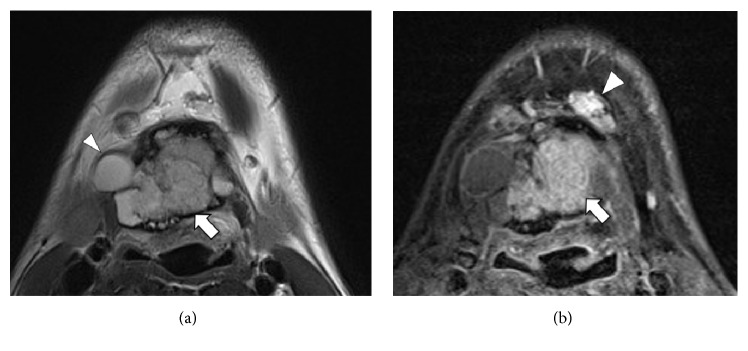
MRI of the neck. (a) T2-weighted image at the level of the hyoid bone shows intermediate signal intensity corresponding to the solid component (arrow) and high signal intensity corresponding to the cystic component (arrowhead). (b) Contrast-enhanced fat-suppressed T1-weighted image at the same level as (a) shows marked enhancement of the solid component (arrow). Left submental lymphadenopathy is also seen (arrowhead).

Computed tomography (CT) demonstrated a heterogeneous mass at the level of the hyoid bone, 35 × 47 × 38 mm in size, expanding the cortical bone of the hyoid (Figure 1). The mass had a central solid component with calcification and a marginal cystic component. Left submental lymphadenopathy was also seen. The normal thyroid gland was noted at the normal paratracheal region. On magnetic resonance imaging (MRI), the solid component appeared hypointense on T1-weighted images and slightly hypointense on T2-weighted images, with marked enhancement on contrast-enhanced fat-suppressed T1-weighted images (Figure 2). Cystic components were also noted as T1 hypointensities and T2 hyperintensities.

Fine needle aspiration of the mass showed papillary carcinoma. Given the imaging findings of expanded cortical bone in the hyoid and FNA findings, we suspected primary papillary carcinoma originating from ectopic thyroid gland tissue associated with a thyroglossal duct remnant in the hyoid bone.

**Figure 3:**
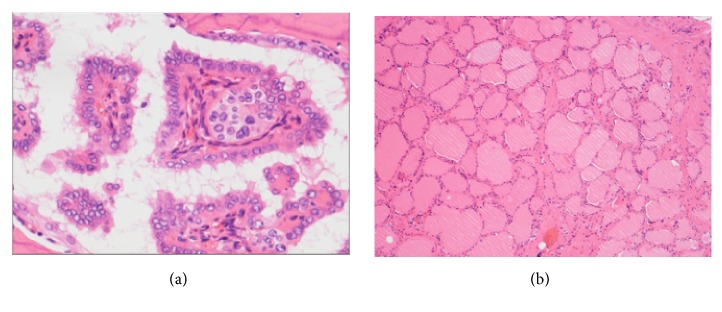
Histopathological finding of the hyoid bone tumor. (a) Microscopically, the tumor comprises atypical cell forming papillae, corresponding to thyroid papillary carcinoma. (b) Thyroid follicles are also seen around the malignant component [hematoxylin-eosin staining; magnification: 400× (a), 100× (b)].

The patient underwent tumor resection and left neck dissection of level I-III. Right neck dissection was not performed, because only left submental lymph node metastasis was suspected from imaging. We usually do not perform preventive neck dissection for thyroid papillary carcinoma in our hospital. The resected specimen showed an ill-defined, whitish mass with calcification measuring approximately 3 cm along the longest axis. Histopathological examination showed papillary adenocarcinoma with calcification (Figure 3). Adjacent tissue invasion such as fibrous stroma, bone, and stratified muscles was seen, but surgical margins were negative. Thyroid follicles were seen around the malignant component. Four lymph nodes (1 peritumoral and 3 left submental lymph nodes) were positive, out of 13 resected lymph nodes. Finally, the patient was diagnosed with papillary carcinoma originating from ectopic thyroid gland in the hyoid bone with multiple lymph node metastases.

Additional thyroid suppression therapy was administered after surgery. The addition of total thyroidectomy and radioactive iodine ablation was not performed to preserve thyroid function because the surgical margins were negative and cancer in the thyroid gland was not suspected from imaging. The patient has been followed up for six months without local recurrence or metastasis.

## 3. Discussion

Carcinomas arising from the anatomical course of the TGD are quite rare. The mean age at presentation of TGD carcinoma is about 40 years. TGD carcinoma tends to be more common in the female population (68.3%). The most common pathology of TGD carcinoma is papillary carcinoma (92.1%) arising from ectopic thyroid tissue, followed by squamous carcinoma (4.3%), which is hypothesized to originate from the wall of the TGD [3]. The clinical presentation of TGD carcinoma is often very similar to that of other benign thyroglossal duct cysts [4]. However, a solid component depicted on imaging, rapid expansion, a fixed cyst, and presence of pain are indicative of malignancy [5].

Imaging findings of TGD carcinoma remain lacking in the literature. It is important to differentiate TGD carcinoma from thyroglossal duct cyst or other benign pathologies. On ultrasound, a mural mass with associated microcalcifications is suggestive of TGD carcinoma [6]. On CT, the presence of a solid component within the course of the TGD raises the suspicion of malignancy but can be also seen in inflammatory processes [7]. Calcification has not been reported in association with thyroglossal duct cyst, even in the presence of chronic inflammation. Calcification on CT thus seems to offer a specific, although not sensitive, indicator of TGD carcinoma [8]. In the present case, the hyoid bone mass had solid and cystic components with calcifications, consistent with the imaging finding of TGD carcinoma.

In the present case, the neck mass was thought to originate in the hyoid bone because the tumor expanded the cortical bone of the hyoid. Differential diagnoses of tumors originating in the hyoid bone vary from benign to malignant lesions. Benign conditions include osteoma, chondroma, aneurysmal bone cyst, and giant cell tumor, and malignant conditions include chondrosarcoma, osteosarcoma, solitary plasmacytoma, and metastatic bone tumors. Most pathologies can be differentiated easily from imaging findings, but excluding the possibility of chondrosarcoma or metastasis by imaging alone is difficult.

TGD is formed along the pathway of normal caudal migration of the thyroid gland. The thyroid gland originates as an invagination of proliferating endodermal cells in the floor of pharynx, which later becomes the foramen cecum in the 3rd to 5th weeks of gestation. The thyroid primordium descends caudally and reaches the paratracheal position in the 7th week of gestation, and then the TGD involutes and atrophies between the 8th and 10th weeks of gestation. The normal anatomic course of the TGD begins at the foramen cecum, then loops around the hyoid bone anteriorly, inferiorly, and posteriorly, and descends anteriorly to the thyrohyoid membrane to reach the normal position anterior to the trachea [1]. Persistent remnants of the TGD may give rise to cysts at any point along the path of descent but are most commonly found inferior to the body of the hyoid bone [8]. TGD uncommonly courses within the hyoid bone but may penetrate the hyoid bone on rare occasions [9, 10]. Penetration of the hyoid bone by the TGD is thought to be a result of forward growth of the hyoid bone, which eventually completely engulfs the duct [10]. This developmental theory supports the development of TGD carcinoma in the hyoid bone.

The standard treatment for TGD carcinoma is a Sistrunk procedure. The addition of neck dissection and total thyroidectomy remains controversial. Neck dissection is necessary when cervical lymph node metastasis is recognized, but preventive neck dissection is not always necessary [3]. In the presence of a normal thyroid gland by clinical or imaging findings, total thyroidectomy in addition to the Sistrunk procedure is unnecessary [11]. In the present case, left neck dissection was performed because submental lymph node metastases were suspected preoperatively. Since cancer in the thyroid gland was not suspected, thyroidectomy was not performed. Thyroid suppression therapy and regular measurement of thyroglobulin has been recommended, although little data supports these measures [12].

## 4. Conclusion

We encountered an extremely rare case of TGD carcinoma originating in the hyoid bone. The TGD uncommonly courses within the hyoid bone but may penetrate the hyoid bone on rare occasions. When we encounter a calcified mass in the hyoid bone, we should consider the possibility of TGD carcinoma as one of the differential diagnoses.
